# The Impact of the Timing of Health-Related Quality of Life Assessments on the Actual Results in Glioma Patients: A Randomized Prospective Study

**DOI:** 10.3390/cancers12082172

**Published:** 2020-08-05

**Authors:** Marthe C.M. Peeters, Hanneke Zwinkels, Johan A.F. Koekkoek, Maaike J. Vos, Linda Dirven, Martin J.B. Taphoorn

**Affiliations:** 1 Department of Neurology, Leiden University Medical Center, 2333 ZA Leiden, The Netherlands; J.A.F.Koekkoek@lumc.nl (J.A.F.K.); L.Dirven@lumc.nl (L.D.); m.taphoorn@haaglandenmc.nl (M.J.B.T.); 2 Department of Neurology, Haaglanden Medical Center, 2262 BA The Hague, The Netherlands; h.zwinkelsvan.vliet@haaglandenmc.nl (H.Z.); m.vos@haaglandenmc.nl (M.J.V.)

**Keywords:** glioma, health-related quality of life, questionnaire, timing, time window

## Abstract

Background: The aim of this study was to explore the impact of the timing of Health-Related Quality of Life (HRQoL) measurements in clinical care on the obtained HRQoL scores in glioma patients, and the association with feelings of anxiety or depression. Methods: Patients completed the European Organisation for Research and Treatment of Cancer (EORTC)’s Quality of Life Questionnaires (QLQ-C30 and QLQ-BN20), and the Hospital Anxiety and Depression Scale (HADS) twice. All patients completed the first measurement on the day of the Magnetic Resonance Imaging (MRI) scan (*t* = 0), but the second measurement (*t* = 1) depended on randomization; Group 1 (*n* = 49) completed the questionnaires before and Group 2 (*n* = 51) after the consultation with the physician. Results: median HRQoL scale scores on t0/t1 and change scores were comparable between the two groups. Between 8–58% of patients changed to a clinically relevant extent (i.e., ≥10 points) on the evaluated HRQoL scales in about one-week time, in both directions, with only 3% of patients remaining stable in all scales. Patients with a stable role functioning had a lower HADS anxiety change score. The HADS depression score was not associated with a change in HRQoL. Conclusions: Measuring HRQoL before or after the consultation did not impact HRQoL scores on a group level. However, most patients reported a clinically relevant difference in at least one HRQoL scale between the two time points. These findings highlight the importance of standardized moments of HRQoL assessments, or patient-reported outcomes in general, during treatment and follow-up in clinical trials.

## 1. Introduction

Gliomas are the most common malignant primary brain tumors in adults, and although rare—a yearly incidence of six cases per 100,000 persons [1]—these tumors have a disproportionate share in morbidity. Glioma patients suffer from both cancer, with a dismal outcome, and a progressive neurological disease. Patients experience symptoms such as headaches, seizures, focal and/or neurocognitive deficits, and changes in personality and behavior [2], which may subsequently negatively influence their Health-Related Quality of Life (HRQoL) [3,4,5,6]. HRQoL is a multidimensional concept covering physical, psychological and social domains, as well as symptoms induced by the disease and its treatment [7]. Both the tumor and its treatment may affect the functioning and well-being of patients [8]. This resulted in patient-reported outcomes such as HRQoL becoming more important in recent decades, besides traditional outcomes such as survival and tumor response on imaging, as they are valuable in evaluating the clinical benefit of a (antitumor) treatment strategy [9]. Indeed, for glioma patients the quality of survival is considered at least as important as the quantity of survival [10]. Furthermore, measuring a patient’s functioning and well-being is an essential part of an integrated approach to disease management. Several instruments are available to assess a patients’ HRQoL, including the Functional Assessment of Cancer Therapy–Brain (FACT-Br) [11], the MD Anderson Symptom Inventory for Brain Tumor (MDASI-BT) [12] and the European Organisation for Research and Treatment of Cancer (EORTC) Quality of Life Questionnaire (QLQ-C30), which can be complemented with a brain tumor module, the EORTC QLQ-BN20 [13].

Appropriate timing of Patient-Reported Outcome Measures (PROMs) is important, particularly in clinical trials, as results should reflect the impact of the treatments under investigation and should not be an effect of timing [14]. For example, if HRQoL is measured during the immediate toxicity of the treatment in one arm and two weeks later in the other arm, when the toxicity effect has faded, erroneous conclusions on the impact of treatment would be drawn. Recommendations about the appropriate timing of PROMs have been formulated, including specifying a standardized moment of questionnaire delivery (e.g., before/whilst/after seeing a clinician). As deviation from these scheduled assessments is likely in practice, a time window needs to be specified that allows questionnaires to be included in the analysis when completed within this window [15,16]. Currently, in trials with glioma patients the predetermined time window differs from 1 week to 6 months [17,18]. However, Ediebah et al. found that the definition of a time window has an impact on the obtained HRQoL results of a study and could alter conclusions about treatment effects [19]. Particularly the width of the time window seems important; a wider completion time window for HRQoL assessments during treatment produced statistically and clinically significant differences compared to a narrow time window [20]. 

Typically, HRQoL questionnaires are administered to glioma patients during follow-up right before their scheduled appointment with the physician to discuss the results of the Magnetic Resonance Imaging (MRI) scan and further treatment. However, at that moment the patient might be suffering from anxiety or may experience feelings of fear for possible progressive disease, which may negatively influence HRQoL scores. Likewise, if administered after the consultation with the physician, feelings of depression or relief might influence the HRQoL scores, depending on the outcome of the consultation. An alternative moment would be to administer the questionnaires at the day of the MRI, which is typically a few days to a week before the consultation with the physician. It is currently unknown what the optimal timing of HRQoL assessments is, and whether assessments at different time points, although within a prespecified time window, would result in different outcomes. The aim of this study was to explore if HRQoL scores changed to a clinically relevant extent when administered between the moment of the MRI scan and the day of the consultation with the physician, and whether feelings of anxiety or depression had an influence on these HRQoL scores.

## 2. Results

### 2.1. Patient Population

Table 1 shows the sociodemographic and clinical characteristics of the 100 participating patients (*n* = 49 in Group 1 and *n* = 51 in Group 2). There were no significant differences between the two groups. The majority was male (58/100, 58%) with a mean age of 56 years (Standard Deviation (SD) 12). The median time since diagnosis was 26 months (interquartile range 9–82 months). Forty-three percent of patients had a glioblastoma, 87% had stable disease and 45% a median Karnofsky Performance Status (KPS) of 90. The mean time between the first and second HRQoL assessment was 7 days (SD = 5), and a few HRQoL and HADS scales were not completed in three patients, representing <1% of all data. 

### 2.2. HRQoL Scores

**Figure 1:**
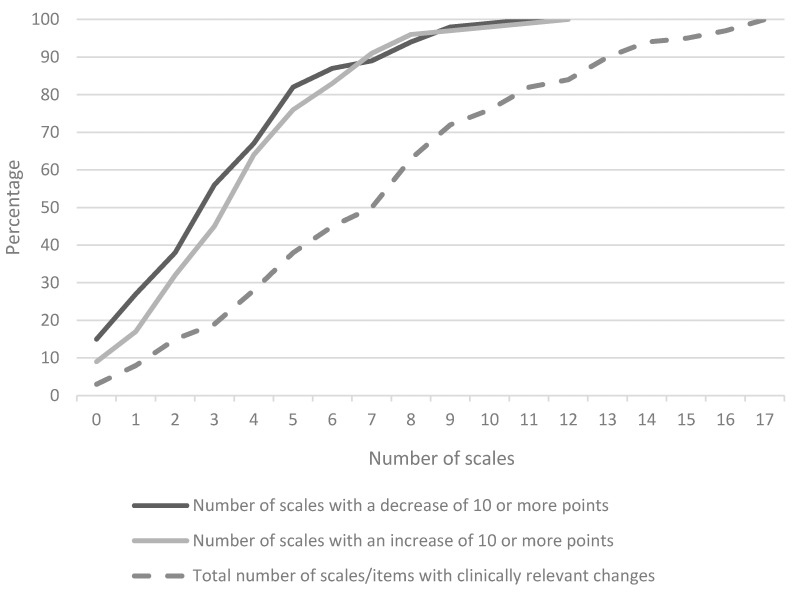
The cumulative percentage of patients with a clinically relevant change in a HRQoL scale, reflecting how the percentage of patients with a change in a specific number of scales add together. Results are shown for the total number of HRQoL scales (dashed line), but also separately for the scales showing a clinically relevant deterioration or improvement.

Median HRQoL scores on the first measurement moment (t0; at the day of the MRI) and the second measurement moment (t1) were comparable between the two groups (Table S1), except for pain on t0, where Group 1 scored significantly lower (median 0 (range: 0–83) vs. median 0 (range: 0–100), *p* = 0.049). On the group level, in both groups, we found that mean changes in HRQoL from t0 to t1 were stable (<10-point change from baseline) for all HRQoL scales. However, at the individual patient level, we found that a large proportion of patients did report a clinically meaningful improvement or deterioration in certain scales, although the percentages varied across scales (Table 2). There were no significant differences between the two groups with respect to the percentages of patients improving or deteriorating to a clinically relevant extent. Therefore, in the next analyses, all patients participating in the study (*n* = 100) were combined. Percentages of patients that changed to a clinically relevant extent ranged between 8–58% for the evaluated scales, with only three patients (3%) remaining stable on all scales. Twelve patients (out of 97) did not deteriorate (i.e., they only reported stable or improved scores) and six patients did not improve (i.e., they reported stable or deteriorated scores). The mean number of the 26 evaluated HRQoL scales that changed to a clinically relevant extent per patient was 7 (SD = 4) (see Figure 1).

In univariable logistic regression analyses, only the KPS score and currently receiving antitumor treatment (no/yes) were predictive (*p* < 0.1) of patients who reported a clinically meaningful change in at least three scales (see Table 3). In multivariable analyses, both a lower KPS (Odds ratio (OR) 0.92, 95% CI: 0.85–0.99) and current antitumor treatment (OR 4.64, 95% CI: 1.18–18.17) were found to be independently predictive of reporting clinically relevant changes (either improvement or deterioration) in three or more HRQoL scales. Results were similar when a cut-off with four or five scales was used. 

### 2.3. Impact of Anxiety and Depression

There were no significant differences between the anxiety and depression scores between the two groups on both measurement moments (Table S1), as well as the change scores from t0 to t1 (Table 2). We evaluated whether a change in the level of anxiety or depression was associated with a change in HRQoL scores using univariable regression. We found that patients that were stable in role functioning had a lower anxiety change score from t0 to t1 (OR 0.781, 95% CI: 0.619–0.984, *p* = 0.036) compared to patients who had a clinically meaningful change, however, not on the other scales. However, this association was no longer significant in the multivariable regression analysis when corrected for confounding factors (OR 0.828, 95% CI: 0.624–1.098, *p* = 0.190). The depression change score was not associated with a change on any of the HRQoL scales, in either group. 

Furthermore, Table 3 shows that both the mean anxiety and depression change scores were not different between patients classified as experiencing clinically meaningful changes (i.e., in ≥3 scales) versus those who were not (i.e., <3 scales).

## 3. Discussion

This study aimed to explore if HRQoL scores changed to a clinically relevant extent between two time points: during the routine MRI scan and the subsequent consultation with the physician. In particular, it was investigated whether the timing of the second assessment was also important, as feelings of anxiety and depression may impact on how patients report their HRQoL. We found that, on the group level, there were no significant differences in any of the HRQoL scores between patients completing the EORTC QLQ-C30 and QLQ-BN20 questionnaires either before or after the consultation with their treating physician, nor in changes in any of the HRQoL scale scores over time. However, on the individual level, we found that there were considerable differences between and within patients. In only 3% of the patients, we did not observe clinically relevant changes on any of the EORTC scale scores, whereas in the large majority the number of scales with clinically meaningful changes ranged from 0 to 17 (mean of 7). Within patients, some of these clinically meaningful changes concerned improvements and others a deterioration, indicating that not all dimensions of HRQoL are affected equally. Possibly, these changes are affected by the patient’s health status, as patients with a better KPS and patients without current antitumor treatment changed on less HRQoL scales, suggesting that health status is of influence. One other study investigating the changes in HRQoL on the individual patient level in glioma patients also found that the majority (84%) of patients showed both deterioration and improvement between two time points [21]. The time between assessments in this study was at least one month and included the initiation of antitumor treatment, which may explain the observed change in HRQoL on the individual patient level. In our study, on the other hand, the two time points were only one week apart, and it is not expected that patients change by a clinically relevant extent within that week if they have a stable health status. Indeed, patients in our study did not initiate treatment in that one-week period, nor did they report a clinical deterioration. In addition, a one- or two-week time period is often chosen in studies that develop a new questionnaire to determine the reliability of that questionnaire (with a test–retest), as patients are not expected to change within that period.

The finding on the group level is in line with the literature in glioma patients, showing that there were no clinically meaningful changes in mean HRQoL scores between different moments of questionnaire administration in situations where the health status of patients was considered not to change significantly, as was the case in our study, where treatment was not altered during this period [22].

This study emphasizes that the time windows of Patient-Reported Outcome (PRO) assessment in clinical trials should be carefully considered, but the exact timing in the disease trajectory may be of importance as well. Although we did not find a relation with anxiety and depression, the period around the MRI and the subsequent consultation may be burdensome for patients. A relatively more stable period, without MRI scans, and changes in treatment or consultations could be considered for the administration of PROMs, although this may be practically challenging. Using a web-based PRO data collection system could facilitate timely evaluation of PROs during the conduct of a clinical trial. 

The changes in HRQoL scores in this study were not influenced by the level of anxiety or depression patients experienced. This is different from a study on patients with a primary diagnosis of recurrent or metastatic non-small cell lung cancer, showing that “scanxiety” is a true phenomenon, resulting in a statistically significant association between greater scan-associated distress and impaired emotional well-being [23]. Furthermore, in our sample only 10 patients (10%) had either progression or (pseudo)progression as an outcome of their MRI scan, equally distributed over the two groups. This small number of patients with (pseudo)progression could be an explanation for the fact that there were no differences in HADS scores at both time points, as well as in the change scores between patients with and without (pseudo)progression. Moreover, we found no differences in any HRQoL scale scores at t0 and t1 or the change scores between patients with and without (pseudo)progression. However, all patients with (pseudo)progression reported clinically meaningful changes in three or more scales, therefore the influence of progression remains unclear. 

This study has several strengths. First, this was a randomized prospective study, which allowed investigation of the impact of the timing of the second administration of HRQoL questionnaires on the reported HRQoL scores. Due to the collection of data at regularly scheduled medical visits, as recommended [24], and because of the short time period between the two assessments, there was almost no missing data. Although we do not have information on the patient characteristics of those not participating (i.e., selection bias), our sample of glioma patients was heterogenous and therefore seems representative of the general glioma population, ensuring generalizability of our results. The choice of a clinically relevant difference, i.e., ≥10 points on a scale, may also have impacted our results. Although this value is universally accepted as a clinically meaningful change and used in cancer clinical trials, recent research has shown that this value may be different for different cancers and may not be applicable to changes on the individual patient level [25,26,27]. More appropriately defined clinically relevant differences may therefore be useful in both clinical trials and practice, when evaluating the impact of treatment over time. 

## 4. Materials and Methods

### 4.1. Study Design and Patients

This was a randomized prospective study for which adult patients (≥18 years of age) with a histologically confirmed grade II-IV glioma (WHO 2016 classification criteria) or radiologically suspected glioma were recruited. Patients were eligible if they did not show progression on previous imaging and were scheduled for a follow-up MRI and corresponding visit to the outpatient clinic. Eligible patients were recruited in Haaglanden Medical Center in The Hague, The Netherlands, between July 2016 and July 2018. The study was conducted in accordance with the Declaration of Helsinki. A declaration of no-objection was granted by the medical ethical review board of the institution (METC Zuidwest Holland, ethic code ‘2016-062’), and all patients provided written informed consent prior to participation. 

### 4.2. Tools

HRQoL was assessed with the EORTC QLQ-C30 [28] and QLQ-BN20 [22]. The EORTC QLQ-C30 is a generic questionnaire developed to measure HRQoL in cancer patients, and comprises five functional scales, one global health status/QOL scale and six single-item scales. The QLQ-C30 was supplemented with the brain-specific questionnaire, the EORTC QLQ-BN20, which includes 20 items assessing four functional scales and seven single-item scales. Both questionnaires are available in Dutch. For functional scales and the global health status/QOL scale, a higher score represents better functioning. For the symptom scales, a higher score means more problems/symptomatology. All single-item and multi-item scales were scored on a 4-point Likert scale, except for the items “overall health” and “overall quality of life” which were scored on a 7-point Likert scale, and subsequently linearly transformed to 0–100 scales. Mean differences of at least 10 points were considered clinically relevant [29].

The Hospital Anxiety and Depression Scale (HADS) is a test of psychological wellbeing and consists of two subscales: one for anxiety and one for depression, each consisting of seven questions. Each question was rated by the patient on a four-point scale, representing the degree of distress suffered by the patient (0 = none, 3 = unbearable). Items for each subscale were summated (range from 0 to 21) and a score of ≥11 on either subscale represented a definite case of anxiety and/or depression. 

The sociodemographic and clinical characteristics were collected using medical records and a study-specific questionnaire.

### 4.3. Randomization and Timing of Assessments

**Figure 2:**
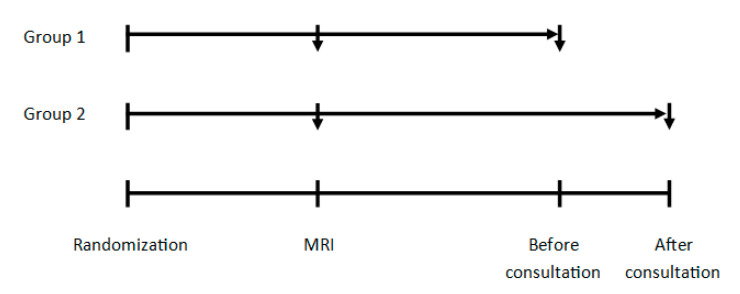
Overview of HRQoL measurements after randomization in glioma patients in Group 1 (assessment on the day of the MRI scan and before the consultation with the physician) and Group 2 (assessment on the day of the MRI scan and after the consultation with the physician).

Patients were randomized into one of two groups (1:1 ratio). Both groups completed the HADS and HRQoL questionnaires at baseline, but the timing of the second measurement differed. Group 1 completed the questionnaires for the second time before the consultation with the physician, and Group 2 directly after the consultation with the physician to discuss the MRI results (see Figure 2 for an overview). As it was impossible to blind patients and nurses to group allocation, the latter were encouraged not to discuss the possible impact of the timing of the HRQoL assessments on the actual HRQoL scores with the patients. Questionnaires were handed out on paper by the nurse-specialist (HZ).

### 4.4. Statistical Analysis 

Descriptive statistics were used to report HRQoL scores as well as the sociodemographic and clinical variables. Means and standard deviations or medians and ranges were calculated for continuous variables, depending on the distribution of the variable. Frequencies and percentages were calculated for nominal variables. Dependent on the type of variable and its distribution, a Student *t*-test, the Mann–Whitney U test, or Chi-square test, were used to compare characteristics between groups. In addition, the percentage of patients whose HRQoL scores had decreased/increased ≥10 points between assessment times were computed, as well as the percentage of patients whose scores remained stable (<10 points change).

We analyzed whether the timing of the second measurement had an impact on HRQoL change scores (comparing change scores between Groups 1 and 2), while adjusting for potential confounding factors (age, sex, KPS, disease status, current antitumor treatment (yes/no)), and baseline HADS and HRQoL scores, by means of Analysis of Covariance (ANCOVA). To examine the determinants of clinically relevant changes in scale scores, logistic regression analyses were used. First, univariable models were constructed assessing which patient- and treatment-related characteristics were predictive of experiencing a clinically relevant change in at least three scales. Associations with a *p*-value ≤ 0.1 were subsequently included in a stepwise backward conditional multivariable logistic regression model. Sensitivity analyses were performed using ≥4 or ≥5 scales, showing a clinically relevant change as cut-off (instead of ≥3 scales as used in the primary analysis). All data were analyzed using the SPSS statistical package (version 25, SPSS, Chicago, IL, USA). The level of statistical significance was set at 0.05 for all analyses. Due to the explorative character of this study, we did not correct for multiple testing.

## 5. Conclusions

In conclusion, the present study found that 97% of patients reported clinically meaningful changes in different aspects of HRQoL in the period between the MRI and the consultation, irrespective of the outcome of imaging. Therefore, time windows for the assessment of HRQoL, or PROMs in general, should be small and centered around standardized moments during treatment or follow-up.

## Figures and Tables

**Table 1:** Baseline sociodemographic and clinical characteristics of glioma patients participating in a randomized trial showing the impact of timing of Health-Related Quality of Life (HRQoL) measurements.

Variable	All (*n* = 100)	Group 1 (Before Consultation) *n* = 49	Group 2 (After Consultation) *n* = 51	*p*-Value
Age, years; mean (SD)	56 (12)	56 (12)	55 (13)	0.855
Sex; n (%) male	58 (58%)	26 (53%)	32 (63%)	0.418
Time since diagnosis (months) (median, range)	26 (6–298)	34 (6–298)	23 (7–256)	0.274
Time between HRQoL assessments (days) (mean, SD)	7 (5)	6 (4)	7 (6)	0.152
Tumor type; n (%)				0.447
Non-glioblastoma	57 (57%)	30 (61%)	29 (57%)	
Glioblastoma	43 (43%)	19 (39%)	22 (43%)	
KPS; median (range)	90 (60–100)	80 (60–100)	90 (60–100)	0.076
Radiological response on MRI; n (%)				0.494
Minor response	3 (3%)	1 (2%)	2 (4%)	
Stable disease	87 (87%)	42 (86%)	45 (88%)	
Progressive disease	9 (9%)	6 (12%)	3 (6%)	
Pseudoprogression	1 (1%)		1 (2%)	
Hemisphere; n (%)				0.34
Left hemisphere	47 (47%)	23 (47%)	24 (47%)	
Right hemisphere	51 (51%)	24 (49%)	27 (53%)	
Both hemispheres	2 (2%)	2 (4%)		
Prior antitumor treatment (multiple options possible); n (%) (maximum *n* = 99)				0.788
Biopsy	14 (14%)	6 (13%)	8 (16%)	
Resection	85 (86%)	42 (88%)	43 (84%)	
Chemotherapy	74 (75%)	38 (78%)	36 (71%)	
Radiotherapy	91 (92%)	44 (90%)	47 (92%)	
Current antitumor treatment; n (%)				0.871
No active treatment	48 (48%)	23 (47%)	25 (49%)	
Chemotherapy	46 (46%)	23 (47%)	23 (45%)	
Other	6 (6%)	3 (6%)	3 (6%)	
Marital status; n (%)				0.31
Without partner	19 (19%)	7 (14%)	12 (12%)	
With partner	81 (81%)	42 (86%)	39 (76%)	
Dexamethasone; n (%)				0.521
Yes	10 (10%)	6 (12%)	4 (8%)	
No	90 (90%)	43 (88%	47 (92%)	
Antiepileptic drug; n (%)				0.227
Yes	56 (56%)	24 (49%)	32 (63%)	
No	44 (44%)	25 (51%)	19 (37%)	
Level of education; n (%)				0.83
Lower	31 (31%)	16 (33%)	15 (29%)	
Higher	69 (69%)	33 (67%)	36 (71%)	

SD: standard deviation, KPS: Karnofsky Performance Status, Level of education: Lower educational status includes primary school, lower secondary school, upper secondary school and post-secondary, non-tertiary school; higher level of education includes short cycle tertiary, bachelor or equivalent, master or equivalent and doctoral or equivalent.

**Table 2:** Mean Changes in HRQol Scale Scores and HADS Scores Between T0 and T1, Between Group 1 and Group 2, and The Number (And Percentage) of Patients with A Clinically Relevant Change Score (I.E. Ten Or More Points).

Scale	Mean Change Scores (95% CI of the Difference) of the Total Group (*n* = 100)	Unpaired *t*-test Mean Change Scores	Mean (SD) Change (t0 to t1) in HRQoL Group 1	Mean (SD) Change (t0 to t1) in HRQoL Group 2	*p*-value for Change (t0 to t1) between Group 1 and 2	Number of Patients in the Total Group (*n* = 100) with a Change Score of <10 or >10 Points, n (%)
QLQc30: Global health status	2.0 (−3.4–7.5)	0.464	−2.3 (14.8)	−4.4 (12.7)	0.840	14 (27.5%)
QLQc30: Physical functioning	−0.4 (−3.9–3.0)	0.798	−1.4 (9.7)	−0.9 (7.5)	0.968	8 (15.7%)
QLQc30: Role functioning	−1.2 (−9.8–7.4)	0.783	−5.4 (21.6)	−4.2 (21.6)	0.414	22 (43.1%)
QLQc30: Emotional functioning	−2.0 (−8.0–4.0)	0.504	−1.5 (15.9)	0.5 (14.2)	0.563	11 (21.6%)
QLQc30: Cognitive functioning	0.6 (−7.7–8.8)	0.893	−2.3 (19.2)	−2.9 (22.0)	0.667	29 (56.9%)
QLQc30: Social functioning	−1.6 (−9.2–6.1)	0.684	−6.8 (20.9)	−5.2 (17.5)	0.903	22 (43.1%)
QLQc30: Fatigue	3.1 (−2.6–8.8)	0.281	7.5 (15.1)	4.4 (13.7)	0.348	28 (54.9%)
QLQc30: Nausea and vomiting	−0.3 (−3.8–3.3)	0.884	1.7 (7.8)	2.0 (9.8)	0.938	7 (13.7%)
QLQc30: Pain	−1.4 (−8.1–5.4)	0.693	−1.0 (18.1)	0.3 (15.8)	0.531	19 (37.7%)
QLQc30: Dyspnea	0.8 (−6.1–7.6)	0.827	2.7 (17.8)	2.0 (16.9)	0.880	10 (19.6%)
QLQc30: Insomnia	0.7 (−6.7–8.1)	0.845	2.0 (19.7)	1.3 (17.6)	0.681	14 (27.5%)
QLQc30: Appetite loss	1.5 (−5.8–8.8)	0.681	5.4 (19.7)	3.9 (17.2)	0.782	11 (21.6%)
QLQc30: Constipation	5.4 (−2.3–13.1)	0.165	4.8 (22.6)	−0.65 (15.6)	0.292	11 (21.6%)
QLQc30: Diarrhea	−1.4 (−7.4–4.7)	0.655	−1.4 (15.1)	0 (15.1)	0.386	7 (14%)
QLQc30: Financial difficulties	4.1 (−3.7–11.8)	0.301	3.4 (17.0)	−0.65 (21.6)	0.363	10 (19.6%)
QLQBN20: Future uncertainty	1.1 (−3.7–5.8)	0.655	3.9 (10.9)	2.8 (12.9)	0.387	18 (36%)
QLQBN20: Visual deficits	3.5 (−0.4–7.4)	0.076	2.4 (8.3)	−1.1 (10.8)	0.142	19 (39%)
QLQBN20: Motor dysfunction	0 (−5.4–5.3)	0.992	−1.4 (14.6)	−1.3 (12.0)	0.754	25 (50%)
QLQBN20: Communication deficit	1.6 (−4.7–8.0)	0.615	2.9 (12.8)	1.3 (18.5)	0.411	20 (40%)
QLQBN20: Headache	0.7 (−7.6–9.1)	0.862	3.4 (23.8)	2.7 (17.6)	0.834	11 (22%)
QLQBN20: Seizures	−5.4 (−11.6–9)	0.090	−2.0 (12.6)	3.3 (18.1)	0.153	4 (8%)
QLQBN20: Drowsiness	−1.3 (−10–7.4)	0.768	2.0 (24.9)	3.3 (18.1)	0.864	15 (30%)
QLQBN20: Hair loss	0.7 (−6.6–7.9)	0.859	−0.7 (17.3)	−1.3 (19.0)	0.600	7 (14%)
QLQBN20: Itchy skin	0 (−6.6–6.6)	0.997	0.7 (18.6)	0.7 (14.2)	0.802	6 (12%)
QLQBN20: Weakness of legs	3.3 (−3.1–9.8)	0.304	0.7 (18.6)	−2.7 (13.2)	0.241	8 (16%)
QLQBN20: Bladder control	1.5 (−4.2–7.1)	0.608	3.4 (15.7)	2.0 (12.4)	0.935	7 (14%)
HADS: Anxiety score	0.1 (−7–9)	0.893	0.2 (2.2)	0.1 (1.5)	0.792	
HADS: Depression score	0.8 (0–1.6)	0.060	1.0 (2.4)	0.3 (1.7)	0.105	

**Table 3:** Univariable and Multivariable Logistic Regression of Associations Between Clinical Characteristics and Patients with A Change of Ten or More Points on Three or More HRQol Scales.

	Univariable Regression		Multivariable Regression	
Variable	*p*-Value	OR, 95%CI	*p*-Value	OR, 95%CI
Current antitumor treatment			0.028	4.636 (1.183–18.170)
No	0.013	Ref		
Yes		5.444 (1.431–20.716)		
Age (years)	0.36	1.020 (0.977–1.065)		
Sex				
Male	0.463	Ref		
Female		1.542 (0.485–4.898)		
Educational level				
Low	0.694	Ref		
High		0.781 (0.228–2.678)		
Partner				
No	0.547	Ref		
Yes		0.615 (0.127–2.990)		
KPS	0.016	0.913 (0.848–0.983)	0.035	0.920 (0.851–0.994)
Disease status				
Stable	0.998	Ref		
Progressive		1.333 (0.457–3.883)		
AED use				
No	0.432	Ref		
Yes		1.556 (0.517–4.682)		
Corticosteroid use				
No	0.644	Ref		
Yes		1.658 (0.194–14.136)		
HADS anxiety change score	0.93	0.987 (0.731–1.331)		
HADS depression change score	0.641	0.940 (0.727–1.217)		

OR: Odds ratio; Level of education: Lower educational status includes primary school, lower secondary school, upper secondary school and post-secondary, non-tertiary school; higher level of education includes short cycle tertiary, bachelor or equivalent, master or equivalent and doctoral or equivalent. KPS: Karnofsky Performance Status; AED: Anti-Epileptic Drugs; HADS anxiety: Hospital Anxiety and Depression Scale anxiety score; HADS depression: Hospital Anxiety and Depression Scale depression score.

## Supplementary Materials

The following are available online at https://www.mdpi.com/2072-6694/12/8/2172/s1, Table S1: Mean scores of HRQoL scales in glioma patients measured before (group1) or after (group 2) the consultation with the physician.
