# Supplementary material for: The Impact of the Timing of Health-Related Quality of Life Assessments on the Actual Results in Glioma Patients: A Randomized Prospective Study
Source: Cancers (Basel). 2020 Aug 5;12(8):2172. doi: 10.3390/cancers12082172 (PMC7465107; doi:10.3390/cancers12082172)
Supplement: Supplementary file 1 [file cancers-12-02172-s001.zip › cancers-871259-supplementary.docx]

**Table S1.** Mean Scores of HRQoL Scales in Glioma Patients Measured before (group1) or after (group 2) the Consultation with the Physician.

|  |  |  | **T0** | | | | T1 | | | |  |
| --- | --- | --- | --- | --- | --- | --- | --- | --- | --- | --- | --- |
| **Nr.** | **Scale** | **N=** | **Total Median (range)** | **Group 1 Median (range)** | **Group 2 Median (range)** | p–value for difference in HRQoL scores between group 1 and group 2 on t0 | Total Median (range) | Group 1 Median (range) | Group 2 Median (range) | p–value for difference in HRQoL scores between group 1 and group 2 on t1 | ANCOVA group 1 vs group 2 on t1* |
| 1 | QLQc30: Global health status | 100 | 75.0 (16.7–100) | 66.7 (16.7–100) | 75 (41.7–100) | 0.256 | 83.3 (8.3–100) | 75 (8.3–100) | 75 (41.7–100) | 0.059 | 0.270 |
| 2 | QLQc30: Physical functioning | 100 | 86.7 (0–100) | 86.67 (0–100) | 93.3 (13.3–100) | 0.305 | 86.7 (0–100) | 86.7 (0–100) | 93.3 (13.3–100) | 0.206 | 0.770 |
| 3 | QLQc30: Role functioning | 100 | 66.7 (0–100) | 66.67 (0–100) | 66.7 (0–100) | 0.558 | 75 (0–100) | 66.7 (0–100) | 83.3 (0–100) | 0.403 | 0.358 |
| 4 | QLQc30: Emotional functioning | 100 | 83.3 (8.3–100) | 75 (8.3–100) | 83.3 (8.3–100) | 0.082 | 83.3 (0–100) | 75 (0–100) | 83.3 (0–100) | 0.236 | 0.322 |
| 5 | QLQc30: Cognitive functioning | 100 | 83.3 (0–100) | 66.7 (16.7–100) | 83.3 (0–100) | 0.192 | 83.3 (0–100) | 66.67 (0–100) | 83.3 (0–100) | 0.191 | 0.829 |
| 6 | QLQc30: Social functioning | 100 | 83.3 (0–100) | 83.3 (0–100) | 83.3 (16.7–100) | 0.412 | 100 (0–100) | 100 (0–100) | 100 (16.7–100 | 0.552 | 0.633 |
| 7 | QLQc30: Fatigue | 100 | 33.3 (0–100) | 44.4 (0–100) | 33.3 (0–100) | 0.133 | 33.3 (0–88.9) | 33.3 (0–88.9) | 22.2 (0–77.8) | 0.403 | 0.397 |
| 8 | QLQc30: Nausea and vomiting | 100 | 0 (0–100) | 0 (0–66.7) | 0 (0–100) | 0.472 | 0 (0–100) | 0 (0–50) | 0 (0–100) | 0.404 | 0.999 |
| 9 | QLQc30: Pain | 100 | 0 (0–100) | 0 (0–83.3) | 0 (0–100) | 0.049 | 0 (0–83.3) | 0 (0–83.3) | 0 (0–83.3) | 0.092 | 0.746 |
| 10 | QLQc30: Dyspnea | 100 | 0 (0–100) | 0 (0–66.7) | 0 (0–100) | 0.976 | 0 (0–66.7) | 0 (0–66.7) | 0 (0–66.7) | 0.883 | 0.544 |
| 11 | QLQc30: Insomnia | 100 | 0 (0–100) | 0 (0–100) | 0 (0–66.7) | 0.658 | 0 (0–100) | 0 (0–100) | 0 (0–66.7) | 0.615 | 0.887 |
| 12 | QLQc30: Appetite loss | 100 | 0 (0–100) | 0 (0–100) | 0 (0–100) | 0.193 | 0 (0–100) | 0 (0–100) | 0 (0–66.7) | 0.147 | 0.656 |
| 13 | QLQc30: Constipation | 100 | 0 (0–100) | 0 (0–100) | 0 (0–100) | 0.186 | 0 (0–66.67) | 0 (0–66.7) | 0 (0–66.7) | 0.889 | 0.154 |
| 14 | QLQc30: Diarrhea | 99 | 0 (0–66.67) | 0 (0–66.7) | 0 (0–66.7) | 0.170 | 0 (0–100) | 0 (0–66.7) | 0 (0–100) | 0.995 | 0.645 |
| 15 | QLQc30: Financial difficulties | 100 | 0 (0–100) | 0 (0–100) | 0 (0–100) | 0.179 | 0 (0–100) | 0 (0–100) | 0 (0–100) | 0.992 | 0.624 |
| 16 | QLQBN20: Future uncertainty | 99 | 20.8 (0–100) | 25 (0–100) | 16.7 (0–58.3) | 0.126 | 16.7 (0–91.7) | 16.7 (0–91.7) | 16.7 (0–66.7) | 0.091 | 0.823 |
| 17 | QLQBN20: Visual deficits | 99 | 5.6 (0–66.7) | 11.1 (0–66.7) | 0 (0–66.7) | 0.134 | 0 (0–66.7) | 0 (0–66.7) | 0 (0–66.7) | 0.696 | 0.090 |
| 18 | QLQBN20: Motor dysfunction | 99 | 11.1 (0–55.6) | 11.1 (0–55.6) | 0 (0–55.6) | 0.085 | 0 (0–88.9) | 11.1 (0–88.9) | 0 (0–77.8) | 0.111 | 0.799 |
| 19 | QLQBN20: Communication deficit | 99 | 11.1 (0–100) | 11.1 (0–88.9) | 11.1 (0–100) | 0.937 | 11.1 (77.8) | 11.1 (0–77.8) | 11.1 (0–77.8) | 0.780 | 0.518 |
| 20 | QLQBN20: Headache | 99 | 0 (0–100) | 0 (0–100) | 0 (0–66.7) | 0.482 | 0 (0–66.7) | 0 (0–66.7) | 0 (0–66.7) | 0.197 | 0.915 |
| 21 | QLQBN20: Seizures | 99 | 0 (0–100) | 0 (0–66.7) | 0 (0–100) | 0.509 | 0 (0–66.7) | 0 (0–66.7) | 0 (0–33.3) | 0.264 | 0.183 |
| 22 | QLQBN20: Drowsiness | 99 | 0 (0–66.7) | 0 (0–66.7) | 0 (0–66.7) | 0.436 | 0 (0–100) | 0 (0–100) | 0 (0–66.7) | 0.483 | 0.728 |
| 23 | QLQBN20: Hair loss | 99 | 0 (0–100) | 0 (0–100) | 0 (0–100) | 0.992 | 0 (0–100) | 0 (0–100) | 0 (0–100) | 0.783 | 0.596 |
| 24 | QLQBN20: Itchy skin | 99 | 0 (0–100) | 0 (0–100) | 0 (0–100) | 0.550 | 0 (0–100) | 0 (0–66.7) | 0 (0–100) | 0.926 | 0.610 |
| 25 | QLQBN20: Weakness of legs | 99 | 0 (0–100) | 0 (0–100) | 0 (0–33.3) | 0.804 | 0 (0–100) | 0 (0–100) | 0 (0–66.7) | 0.441 | 0.123 |
| 26 | QLQBN20: Bladder control | 99 | 0 (0–100) | 0 (0–66.7) | 0 (0–100) | 0.445 | 0 (0–100) | 0 (0–66.7) | 0 (0–100) | 0.618 | 0.999 |
| 27 | HADS: Anxiety score | 99 | 3 (0–16) | 3.5 (0–16) | 3 (0–9) | 0.193 | 4 (0–17) | 4 (0–17) | 3 (0–9) | 0.117 | 0.920 |
| 28 | HADS: Depression score | 99 | 3 (0–15) | 4 (0–15) | 2 (0–11) | 0.080 | 2 (0–19) | 2 (0–19) | 1 (0–10) | 0.571 | 0.104 |

*Each analysis is corrected for the following variables: score at t0, KPS, MRI outcome, gender, age, anti–tumor treatment and anxiety and depression score
